# Sticking the landing: A comparison of shod vs barefoot landing kinetics and foot muscle characteristics in gymnasts, cheerleaders, and non-athletes

**DOI:** 10.1371/journal.pone.0309157

**Published:** 2024-10-04

**Authors:** Sarah T. Ridge, Dallin I. McLean, Kelsey R. Garner, Mark T. Olsen, Dustin A. Bruening, A. Wayne Johnson

**Affiliations:** Department of Exercise Sciences, Brigham Young University, Provo, Utah, United States of America; The University of British Columbia, CANADA

## Abstract

Objectives: The ability to control landings and stabilize quickly is essential in sports like gymnastics and cheerleading, where landing quality impacts scores. The similarities and contrasts between these sports, where one trains primarily barefoot and the other shod, may increase understanding of the kinetic role of the foot during landings. Design: Sixteen gymnasts (GYM), sixteen cheerleaders (CHR), and sixteen non-athletes (NAT) performed single-foot shod and barefoot drop landings onto a force plate. Method: Foot muscle strength was assessed using a custom test and ultrasound imaging was used to measure six foot muscles. Group differences in muscle sizes and strength measurements were compared using one-way ANOVAs (α = 0.05). Landing mechanics metrics were evaluated using 3 x 2 between-within ANOVAs (α = 0.05). Pairwise comparisons were made using Tukey post-hoc tests. Results: Barefoot landings resulted in greater peak vertical ground reaction force (pVGRF) and lower time to pVGRF (TTpVGRF). Significant group main effect differences were found between GYM and NAT for all kinetic measures (GYM: shorter time to stability (TTS) and TTpVGRF, and greater pVGRF), while no significant differences in landing kinetics were found between CHR and either GYM or NAT. No interactions were found between group and condition. GYM and CHR had significantly greater summed foot muscle size than NAT, however, only CHR displayed significantly greater toe flexion force than NAT. Conclusions: Our data suggests that while wearing shoes does not affect groups differently, footwear reduces initial peak VGRFs but does not influence later stabilization times.

## Introduction

Landings are a critical part of performance in a number of sports and recreational activities. The nature of landings varies both across and even within sports. Controlled landings are particularly important in certain athletic performances, such as gymnastics and cheerleading, in which scores are dependent on the quality of the landing. The ability to stabilize quickly upon landing after an aerial maneuver allows athletes in these sports to score higher on the skill as well as transition to the next element in a routine [1]. Yet, rapid stabilization may run counter to the goal of absorbing high impact forces [2] and may be associated with specific landing strategies and/or muscular adaptations in order to avoid impact-related injuries [3]. Understanding how gymnasts and cheerleaders stabilize upon landing could help in developing training protocols to balance performance improvement with injury prevention.

Gymnastics and cheerleading contain many similarities, but also some important differences.

Tumbling maneuvers (e.g. handsprings, flips, and twists), performed in isolation or in sequence, are necessary components of both events. Furthermore, both gymnasts and cheerleaders perform frequent landings from an elevated height: in gymnastics, off of a beam, vault or bars, while in cheer, off of pyramids and stunts. However, the groups train and perform on different surfaces: gymnasts use sprung floors and cushioning mats, where cheerleaders often train on thin foam (i.e. “dead-mat”) and can perform on grass or hardwood. Additionally, gymnasts train and perform barefoot, while cheerleaders typically train and perform shod. The common characteristics of these sports may lead to similar adaptations in these athletes when compared to the general population. However, the variation in training and performance may also result in unique adaptations between these groups of athletes. Some of these unique adaptations may occur in the foot and lower leg muscles as previous research has shown greater muscle size and/or strength when activities are performed barefoot or in minimalist footwear rather than in structured, cushioned shoes [4–8].

The foot plays a major role in landings, as it is the first point of contact with the ground. While most studies of drop landings and stabilization upon landing have focused on the ankle, knee, and hip joints, those that have examined foot kinematics in landing have found differences with varying drop heights [9], in barefoot vs shod conditions [10], and with varying landing surface stiffness [11]. Previous studies have shown the important role of the foot during landings. For example, the midfoot performs a significant amount of lower extremity work during landings [12], and foot musculature both contributes to the work done at the ankle and midfoot [13] and contributes to jump and landing performance [14]. The similarities and differences in training and performance between gymnasts and cheerleaders presents a unique opportunity to examine landing strategies, stabilization, and foot musculature in a trained population.

To better understand the kinetic role of the foot during landing, time to stability and vertical ground reaction forces during single foot drop landings, as well as foot muscle size and strength, was studied in a group of gymnasts, cheerleaders, and non-athlete control participants. Based on familiarity with landing while barefoot or shod, it was hypothesized that gymnasts would stabilize more quickly than the other groups during barefoot landings, while cheerleaders would stabilize more quickly than the other groups during shod landings. Additionally, it was hypothesized that gymnasts and cheerleaders would have higher peak vertical ground reaction forces than controls in both conditions [15]. Furthermore, due to lack of familiarity performing landings in shoes, gymnasts would have higher peak vertical ground reaction force than cheerleaders in shoes, while the reverse would occur barefoot due to the novelty of cheerleaders landing barefoot. Finally, it was hypothesized that gymnasts would have the largest muscles and greatest foot strength, and controls would have the smallest muscles and lowest foot strength.

## Methods

### Participants

Sixteen female gymnasts (GYM), 16 cheerleaders (CHR), and an age- and mass-matched group of 16 non-athletes (NAT) were recruited from the university student body (Table 1) between 08-03-2016 and 07-03-2017. In order to ensure sport differentiation, all athletes were required to have been training in either gymnastics or cheerleading for at least the last five years and could not have participated in the other activity during the previous five years of training. NAT participants exercised less than 100 min/week and had never participated in high school, collegiate, or intramural sports. NAT participants were recruited last, and were matched to the mean body mass of GYM and CHR through selective recruitment. All subjects were required to be free of lower extremity injuries or any injury that may have affected their balance (nerve injury, concussion, etc.) during the last 6 months, as reported in a questionnaire. All participants were volunteers and signed written Institutional Review Board approved consent forms (Protocol # X16110).

**Table 1:** Participant demographics. Values are mean ± standard deviation.

	CHR	GYM	NAT	p-value (ω^2^)
*N*	16	16	16	---
Age (yrs)	20.4 ± 1.7	19.5 ± 1.1^A^	21.5 ± 1.4	0.002* (0.213)
Height (cm)	161.9 ± 5.4^B^	159.3 ± 4.9^A^	167.2 ± 5.8	<0.001* (0.247)
Mass (kg)	58.7 ± 7.1	56.7 ± 4.3	57.2 ± 5.7	0.779 (0.000)
Foot length (cm)	23.5 ± 1.18	23.3 ± 0.92	23.9 ± 0.74	0.177 (0.032)

* denotes significant main effect

^A^ denotes a significant pairwise difference between GYM and NAT

^B^ indicates a significant pairwise difference between CHR and NAT

### Procedures

All tests were performed in one scheduled visit. Participants were tested at different stations for 1) foot muscle size, 2) foot muscle strength, and 3) drop landing kinetics, in that order. Researchers taking physical measurements were blinded to the participants’ group. Muscle size, foot strength, and single foot landings were all performed on the participant’s preferred foot for kicking a ball.

**Fig 1:**
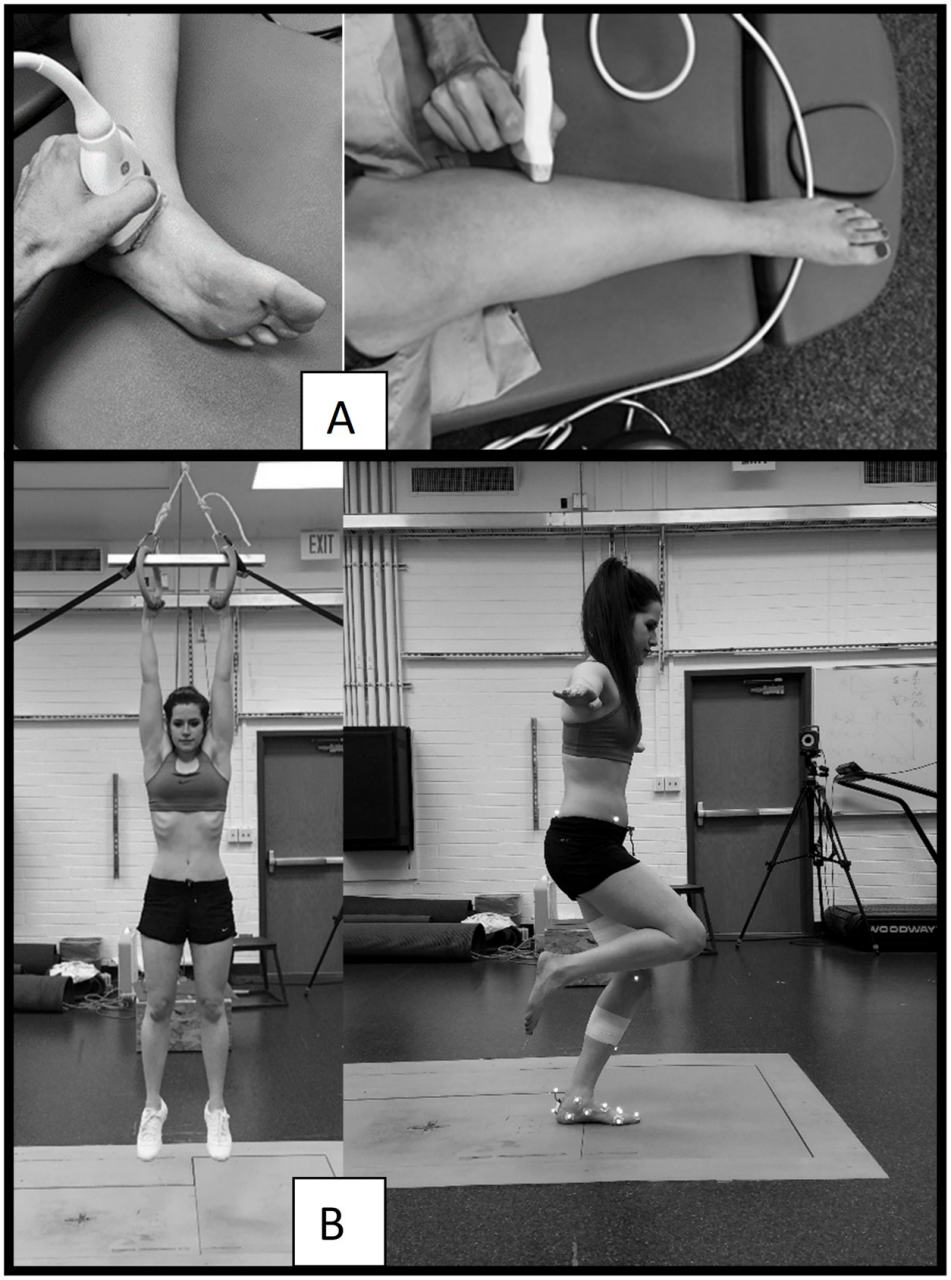
Data collection procedures. A) muscles were imaged using ultrasound, then measured to report muscle sizes, B) drop landing procedures–the image on the left represents the start position, while the image on the right shows the landing of a successful barefoot trial. The individual pictured in this manuscript has given written informed consent (as outlined in PLOS consent form) to publish their image.

Ultrasound imaging was used to assess and collect foot muscle size data (Fig 1a). Six foot muscles that may contribute to landing mechanics [13, 14, 16] and that had previously established measurement protocols were selected: flexor digitorum brevis (FDB), quadratus plantae (QP), abductor hallucis (AH), fibularis brevis (FB), fibularis longus (FL), and tibialis posterior (TP). Measurements were taken using a ML6-15 linear probe or a 9L linear probe (GE LogiqP6, General Electric Company, Fairfield, CT) depending on the muscle being measured. Two images were collected for each muscle by a single experienced ultrasound operator. Intra-rater reliability, measured by ICC, for the ultrasound operator ranged from 0.983–0.998 for each of the muscles measured. Images used for measurement of the AH were taken with the transducer placed transversely across the foot, using the navicular tuberosity as a consistent internal landmark. The transducer was then rotated to the plantar surface of the foot to record images of the FDB and QP. Images for the measurement of the TP and FL were recorded at 30% of the length of the lower leg, measured from the knee joint line to the inferior aspect of the lateral malleolus. The probe was positioned on the anterolateral shank for the TP, then rotated slightly laterally to image the FL. The FB was measured at 50% of the length of the lower leg. More details about collection, measurement, and reliability have been previously published by our lab [7, 17–19].

In this study, maximal toe flexion force generation was used as an indicator of foot muscle strength. Force was measured using a custom-built dynamometer-based apparatus. Separate tests were completed to quantify toe flexion strength from the great toe and lateral toes as the participants gripped a carabiner (great toe) or metal bar (lateral toes). Three trials of each test were performed. Details about collection procedures and reliability of these measurements are available in previous publications [20, 21].

Ground reaction force data were collected as participants performed single foot drop landing trials shod (Nike Cheer Unite) and barefoot, in random order. For each trial, participants hung with straight arms from gymnastics rings positioned so that the distance from their heel to a force plate (OR6 series, AMTI, Inc., Watertown, MA) below was 0.4 m (Fig 1b). This height was chosen to emphasize foot and ankle contribution to the landing phase, as previous studies have observed that landings from heights greater than this increase hip and knee contributions [10, 22]. The participants were instructed to hold onto the rings until directed to let go, land on one foot, then hold their natural landing position for 10 seconds. No instructions were given regarding specific landing techniques. Trials were repeated if the participant did any of the following: 1) lifted herself up or swung on the rings before letting go, 2) landed with part of the foot off of the force plate or stepped off of the force plate before the 10-second trial was complete, or 3) used the opposite foot to restore balance. Three successful trials were collected in each condition. Force data were collected at 1000 Hz.

### Data processing

Cross-sectional areas (CSAs) of each muscle were assessed by manually outlining the internal fascial border of each muscle using the US manufacturer-supplied software [18, 19, 23]. The two measurements were then averaged for statistical analysis.

Strength data were analyzed in custom LabVIEW software (National Instruments, Austin, TX). The highest average force over a stable one-second region was identified in the software, as detailed previously [21]. Peak force measures were averaged from the three trials of each test for use in statistical analyses.

**Fig 2:**
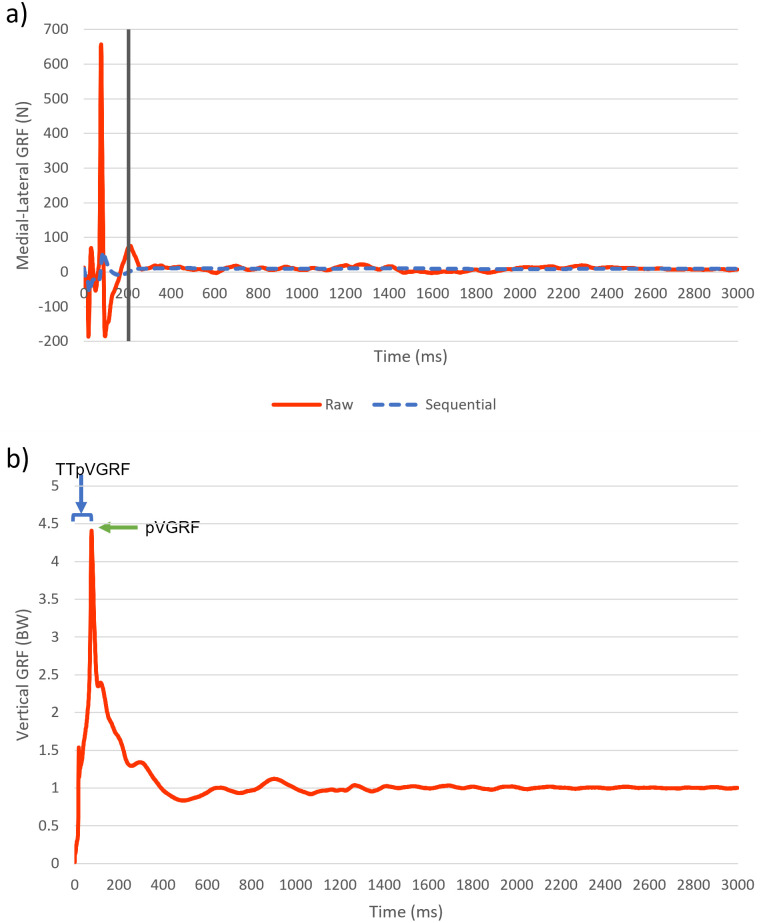
Representative ground reaction force graphs from a barefoot landing. a) Medial-lateral GRF. The solid (red) line represents the raw GRF data. The dotted (blue) line represents the sequential average of the GRF data, from which TTS was calculated. The thick (black) vertical line represents the TTS (when the sequential average of the GRF data reached and subsequently stayed within .25SD of the overall series average.) b) Vertical GRF. The solid (red) line represents the raw GRF data. The arrows show the peak VGRF (pVGRF) and the time interval from landing to pVGRF (TTpVGRF).

All ground reaction force data were analyzed in custom LabView software. Time-to-stability (TTS) was calculated from the medial/lateral forces during the first 3 seconds following initial contact (Fig 2a). A sequential estimation technique was used to find TTS from initial contact until the cumulative average of the signal remained within 0.25 standard deviations of the overall series mean [24]. TTS from ML forces was chosen to emphasize stabilization during the initial loading phase of the landing and the contribution from smaller foot intrinsic and extrinsic muscles. Two metrics were extracted from vertical ground reaction force data: peak force (pVGRF) and time from initial contact to peak force (TTpVGRF) (Fig 2b). Vertical ground reaction force data were normalized by participants’ body weight to facilitate comparisons among groups.

### Statistical analyses

A repeated measures design with Latin square randomization was used to address the hypotheses. Group differences in each muscle size and strength metric were evaluated using one-way ANOVAs (α = 0.05) with Tukey post-hoc tests for pairwise comparisons. Landing mechanics metrics (TTS, pVGRF, TTpVGRF) were evaluated using 3 x 2 between-within ANOVAs (α = 0.05) to evaluate differences among the three groups and between the two conditions. Group pairwise comparisons were made using Tukey post-hoc tests. Multiple comparisons in group main effects were accounted for by running the Benjamini-Hochberg procedure with a false discovery rate of 0.10. This procedure controls the false discovery rate by ranking the p-values and comparing them to a critical value. JASP software (v. 0.12.2) was used for all statistical analyses, except for the Benjamini-Hochberg procedure which was performed using a spreadsheet [25].

## Results

A total of 16 group main effects were statistically analyzed (Table 1, S1 and S2 Tables). The Benjamini-Hochberg procedure indicated that 12 of the 16 p-values were significant, with the highest significant p-value being 0.054.

### Participant demographics

Although groups were matched by mass, statistically significant differences were found between groups in age and height (Table 1). Pairwise comparisons showed that GYM was significantly younger than NAT (p = 0.001), while NAT was taller than both GYM and CHR (p<0.001 and p = 0.023, respectively).

### Landing kinetics

**Fig 3:**
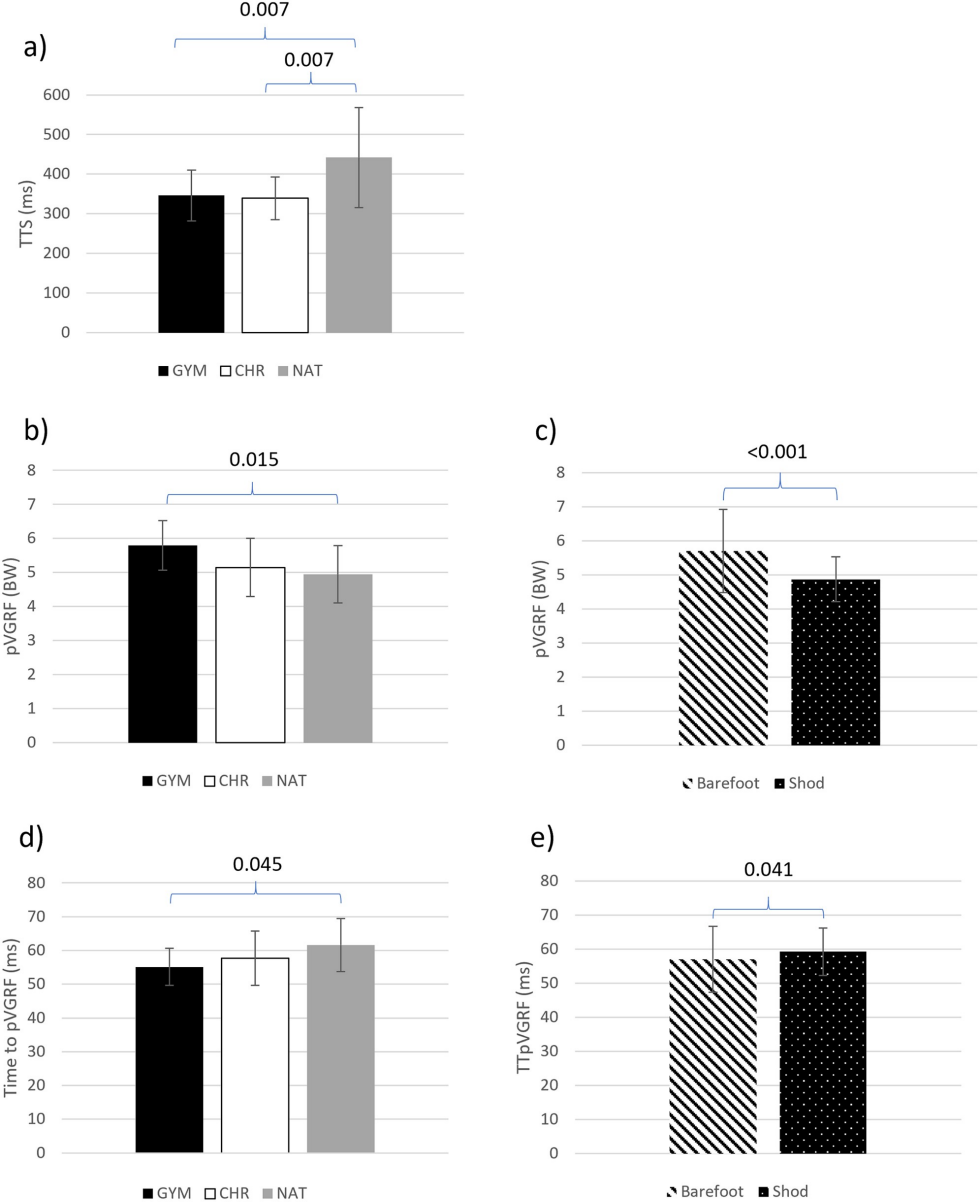
Landing kinetics data representing comparisons which showed statistically significant differences. p-values for the statistically significant differences are included above the appropriate pairs of data. Error bars represent standard deviation. a) group averages for TTS, b) group averages for pVGRF, c) average pVGRF for each footwear condition, d) group averages for TTpVGRF, and e) average TTpVGRF for each footwear condition.

There was a group main effect difference in TTS (p<0.001). Pairwise comparisons showed significant differences for both GYM and CHR compared to NAT, achieving stability 37% and 33% faster than NAT, respectively (Fig 3a). No differences in TTS were observed between GYM and CHR. No significant main effect was observed between barefoot and shod conditions (p = 0.413), nor was there a significant interaction between groups and conditions (p = 0.761).

There was a group main effect difference in pVGRF (p = 0.015). Post hoc comparisons showed differences between GYM and NAT, with GYM displaying approximately 15% greater pVGRF than NAT (Fig 3b). A significant main effect was also found between the barefoot and shod conditions (p<0.001), with all groups displaying greater force values in the barefoot condition (Fig 3c). GYM displayed the greatest difference between conditions, generating a 23% greater peak in the barefoot condition than the shod (S1 Table); however. no significant interaction was found between groups and condition (p = 0.144).

With the Benjamini-Hochberg procedure, TTpVGRF showed significant group main effects (p = 0.054), with GYM showing significantly smaller TTpVGRF than NAT (Fig 3d). A significant difference was shown between the barefoot and shod conditions, all groups achieving peak forces in less time in the barefoot condition (p = 0.041; Fig 3e). The greatest difference between conditions was once again found in GYM, achieving peak forces 7.9% faster when barefoot (S1 Table); however, no significant interaction was found between groups and condition (p = 0.34).

### Muscle size and strength

**Fig 4:**
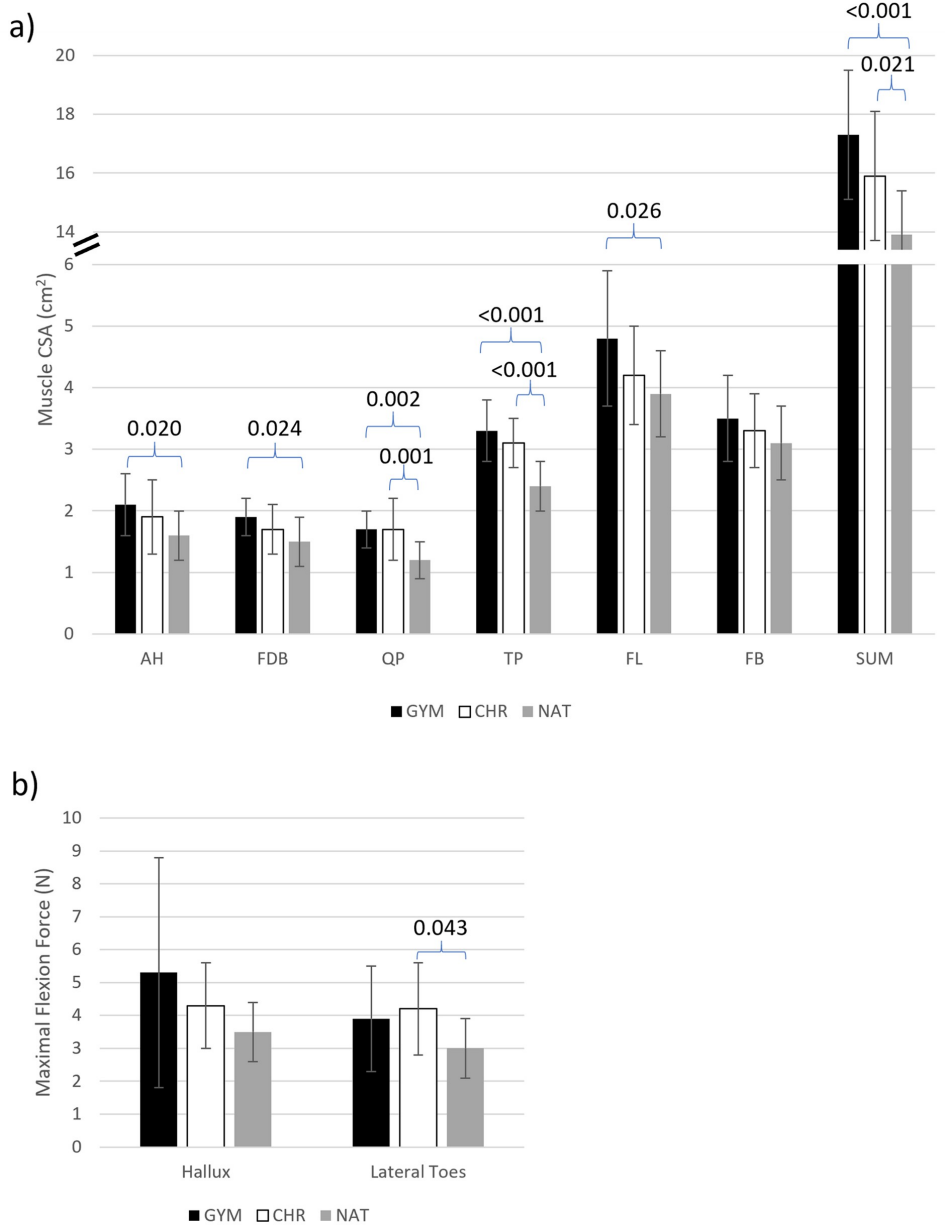
Foot muscle size and strength data for each group. Error bars represent standard deviation. p-values for statistically significant comparisons are included above the appropriate pairs of data. a) average foot muscle size for each group, b) average foot strength for each group.

Main effect differences in muscle size were seen for all muscles but one (AH p = 0.026, FDB p = 0.031, QP p<0.001, TP p<0.001, FL p = 0.025, FB p = 0.296) (S2 Table). Significant pairwise differences were observed between GYM and NAT, while differences between CHR and NAT were found only in the QP and TP (Fig 4a). No significant differences were found between GYM and CHR. A main effect difference was also found for total muscle size (p<0.001) (S2 Table). Pairwise comparisons showed that total muscle size was significantly greater in both GYM and CHR compared to NAT by 24% and 14%, respectively (Fig 4a).

A main effect group difference was found for lateral toe-flexion strength (p = 0.042) (S2 Table). Pairwise comparison showed a significant difference between CHR and NAT in the lateral toes test, with CHR generating 40% more force than NAT (Fig 4b). No significant differences were found between the GYM group and the other groups. There was large variability within the GYM group due to two participants with high, but reasonable, strength measurements (Fig 4b).

## Discussion

The purpose of this study was to investigate differences in landing kinetics, foot muscle size, and foot muscle strength between gymnasts, cheerleaders, and a non-athlete group in both shod and unshod conditions. While similar studies have examined landing characteristics of gymnasts compared to other athletes and to non-athletes, this was the first to include cheerleaders as a similarly trained athletic group. Overall, our hypotheses were partially met: group differences were found in both landing kinetics and in muscle size/strength and condition differences were found in landing kinetics. However, no interactions were found between condition and group in landing kinetics.

### Landing kinetics

#### Time to stability

Our hypothesis that gymnasts would stabilize fastest in the barefoot condition while cheerleaders would stabilize fastest when shod was not supported; the basis of this hypothesis was that the athletes would stabilize fastest in the shoe condition that they habitually train in. Since both athlete groups stabilized faster than NAT whether shod or barefoot, but there was no difference in stabilization time between conditions, our results suggest that rapid stabilization following a drop-landing does not depend on the shoe condition that the athlete is accustomed to training in, but instead on athletic ability. The only other study that has investigated TTS following a landing in different footwear conditions (barefoot and three shoes with varying cushioning) also found that footwear choice had no effect on stabilization time in runners [26].

#### Vertical ground reaction forces

Our hypothesis that GYM and CHR would produce higher pVGRF than NAT was partially met, as a difference was found between GYM and NAT, but not between CHR and NAT. Additionally, our hypothesis that GYM would have higher pVGRF than CHR in shoes was not met, nor did the reverse occur as hypothesized. The high pVGRF values found for GYM compared to NAT are typical of gymnasts, a tendency that has been reported by other researchers which has been attributed to the fact that GYM are used to landing on softer surfaces [15, 27]. As drop height—and, therefore, pVGRF—increases, this difference appears to become even more exaggerated [27]. Though all groups had lower pVGRF in the shod condition, the decrease in pVGRF was similar for all groups. While shoes theoretically provide a cushion that should decrease pVGRF, research on the differences between pVGRF in shod and barefoot landings has been equivocal, with some studies finding increased pVGRF when shod [28, 29], others when barefoot [30, 31], and others finding no difference [32, 33]. The discrepancies likely result from participants changing their mechanics based on landing expectations. For example, Fong Yan et. al. [34] found that the body prepares the lower limbs for impact by adjusting joint stiffness in relation to the perceived hardness of the landing surface. Additionally, differences in the components of study protocols (e.g. footwear type, testing population, takeoff conditions, drop height, landing surface, and landing strategy) may influence the effects of the interactions among all of these factors. Most studies, including ours, have not prescribed the use of a specific landing style, therefore differences in study outcomes may be due to varying mechanical adjustments during the landing.

Generally, an increase in peak forces is accompanied by a decrease in time to peak force or in loading rate. This was true for the current study, as GYM achieved pVGRF fastest, and NAT slowest. These findings have been supported in additional studies [15, 35, 36], with another study that didn’t reach significance [27]. Similarly, all groups had shorter TTpVGRF in the barefoot condition. As with pVGRF, previous literature comparing barefoot and shod landing loading rates is also equivocal [31, 37] and may similarly be due to varying study protocols. With respect to the lack of group by condition interactions in both pVGRF and TTpVGRF, it is also important to note that landings in our study were always toe-to-heel. Therefore, footwear may not have influenced ground reaction forces in the same way that it does in running, where changing footwear often triggers kinematic changes (changing from rearfoot strike to forefoot strike) [38, 39]. Anecdotally, many of the cheerleaders commented that the shoes used felt fairly stiff; a more compliant shoe may have impacted our findings differently. Overall, our results combined with these others suggest the need for additional research exploring the relationships between footwear, population type, and loading rate during various athletic activities.

The characteristically “stiff” landing style demonstrated by gymnasts contribute to high VGRFs and loading rates [40]. It has been proposed that this landing style may be the reason why a large number of injuries occur during gymnastics landings and why the lower limbs account for the greatest proportion of gymnastics injuries [11, 40]. In recent years, judging has changed to allow more freedom of movement during landings (e.g. more knee flexion/ a less “stiff” style) [41]. Epidemiological studies of gymnastics injuries have not focused on landing technique and injury in light of judging changes, though it may be interesting to compare injury rates from post-2017-2018 to previous years.

Decreased injury incidence may also be achieved by training gymnasts to land differently so that they can decrease VGRFs. However, our results suggest that a stiff landing may be beneficial to gymnastic performance, enabling quick stabilization. In other words, gymnasts may sacrifice a soft landing in order to maintain body position that allows them to “stick” landings and transition to other skills. McNitt-Gray et. al. [35] commented that the distinct gymnast landing style may be explained by the need to maintain balance during competitive gymnastics landing. Sabick et. al. [15] also suggested that the increased peak GRF is likely due to adaptations by the athletes to the gymnastics scoring system, in which athletes have traditionally been encouraged to land upright and with relatively straight legs. It has also been suggested that gymnasts develop this distinct landing as a result of the surfaces they train on; that since the surface is compliant, muscles would be pre-activated before landing and the body would be more rigid at impact [42]. Although there have been adjustments to gymnastics judging criteria regarding knee flexion during landing (as suggested by Slater et al. [40]), continued education of judges and coaches may be helpful to reduce injuries. Otherwise, training protocols should focus on strengthening lower limb muscles and joints that are involved in landings while simultaneously decreasing the frequency of high-impact landings.

Generally, stiff landings are not as necessary in cheerleading, since points aren’t awarded for sticking landings, but instead for completing the aerial skill successfully. It is usually acceptable in cheerleading to step out of a tumbling pass, where in gymnastics it is not. Only two previous studies could be found that investigated the kinetics of cheerleading landings, but both were too dissimilar to ours to draw comparisons. The first [43] grouped cheerleaders together with dancers to compare against a group of volleyball and soccer players and a non-athlete group. Athletes were instructed to vertically jump and land as they would in their respective sports. Thus, they did not control jump height, while our study used a drop landing from a standardized height. The other [44] was a small study of 3 flyers during assisted landings following a partner stunt. Each of these landings was assisted by the base cheerleader, who essentially softened the landing of the flyer. Due to the lack of research on landing kinetics of cheerleaders, our study could serve as a baseline reference for future studies.

### Muscle measurements

#### Foot muscle size

While both athlete groups had greater summed foot muscle size than non-athletes, only two individual muscles (QP and TP) were larger in both athlete groups compared to NAT. Both of these muscles assist in eccentrically controlling the medial-longitudinal arch during loading [23, 45, 46]. In particular, the QP is more critical for arch control when the toes are in a closed chain position, as in toe-heel landings, than during gait or other heel-first loading tasks. It is possible that the athlete groups had larger QP and TP muscles than NAT due to their training, including regular tumbling and frequent toe-heel landings.

The greater foot muscle size displayed by the athletes may also relate to the increased VGRFs they experience. With greater peak landing forces, there may be greater eccentric muscle activity and more stress placed on the foot and ankle muscles, stimulating hypertrophy. With increased muscle size, the ability of the foot to absorb the impact forces may be enhanced.

#### Toe flexor strength

Contrary to our hypothesis, these results show that only cheerleaders had greater toe flexor strength than non-athletes. Two previous studies have also found that toe flexor strength is correlated with the CSA of the FDB [47, 48], while Kurihara [48] additionally found correlations with QP, flexor hallucis brevis, and lumbricals. In the current study, no differences were found in toe flexor strength between GYM and CHR. The groups might have been too similar in regards to mass, foot size, and activity-based stresses placed on the foot to bring about unique strength adaptations [49].

### Limitations

As with all human subjects research, there are a few limitations that should be considered when interpreting the results of this study. First, although participants in the NAT group were recruited by matching mass to the athletes in the study, the NAT group was significantly taller than the CHR and GYM groups. While anthropometrics may influence stability, previous research found non-significant, weak-moderate correlations between height and balance in female gymnasts and control participants [50]. It should also be noted that while there were no statistically significant differences between GYM and CHR, the pattern in most of the results was that GYM and NAT were the most different, and CHR was between both, but tending to be much more similar to GYM than to NAT. Because of the similarities between these two sports, many cheerleaders were gymnasts before they took up cheer, and the athletes in both sports are often similar in stature. This was reflected in our subjects, as there were no significant between-group differences in mass or foot length. It is possible that differences between these similar groups are subtle, and it would take a substantially greater number of participants to reveal them. Thus, the clinical interpretation of our results may be slightly different from the statistical results.

To address the inherent limitations often associated with drop landings, a hanging drop method was used rather than a box drop to limit variation in drop heights. Additionally, the chosen drop height was anecdotally challenging as a single barefoot drop onto a hard surface. However, in the shod condition, it may not have been sufficiently challenging to elicit interactions among groups. Therefore, future studies may need to address the complex interactions among the various methodological components.

Strength testing of foot musculature also has inherent challenges since these muscles are difficult to isolate and protocols are only recently being developed [20, 21, 51]. The custom-made dynamometer used has shown high reliability in general adult populations [20, 21]. However, because the athletes tested were relatively smaller people with short toes, some of them had difficulty gripping the toe attachments (both the carabiner and metal bar). Because of this, they may not have been able to consistently give maximum effort during the strength testing, which may have increased variability in the results. Adjustments could be made to the device to make it more suitable for smaller users. In other situations where this device was not suitable, a pressure mat has been used instead with high reliability [19, 21]. In the future, alternate measures of foot strength such as inversion, eversion, and eccentric toe flexion could be included. One muscle (FL) that was found to be significantly larger in GYM vs NAT is an evertor of the subtalar joint and was therefore not involved in our method of strength testing.

## Conclusions

Our data showed differences between groups in measurements of landing kinetics and muscle size/strength. Differences in landing kinetics between landings performed shod and barefoot were found. Although footwear did not affect groups differently, our data supports a growing body of literature showing that wearing shoes can reduce initial peak VGRFs, but does not influence later stabilization times. The shorter stabilization times shown by our athletes may be related to their greater foot muscle size and training. Although gymnasts and cheerleaders were more similar than expected, overall there appeared to be a progression in most kinetic variables, spanning from non-athletes to cheerleaders to gymnasts. The performance constraints of gymnasts in particular may contribute to their high pVGRFs, but may also result in increased risk of injury. Strengthening lower limb muscles and/or decreasing the number of repetitions of high-impact landings may help mitigate the deleterious effects of high pVGRFs.

## Supporting information

S1 Table Landing kinetics. Comparison of time to stability, peak vertical ground reaction force, and time to peak vertical ground reaction forces between conditions (shod and barefoot) and among groups (GYM = gymnasts, CHR = cheerleaders, NAT = non-athletes). Values are mean ± standard deviation. p-values are group main effect, except where specified by a superscript letter which reflect the appropriate pairwise value.
(DOCX)

S2 Table Muscle size and strength. Comparison of muscle cross-sectional area (CSA) and maximal toe flexion force measurements among groups (GYM = gymnasts, CHR = cheerleaders, NAT = non-athletes). Values are mean ± standard deviation. p-values are group main effect, except where specified by a superscript letter which reflect the appropriate pairwise value.
(DOCX)
