# Supplementary material for: Sticking the landing: A comparison of shod vs barefoot landing kinetics and foot muscle characteristics in gymnasts, cheerleaders, and non-athletes
Source: PLoS One. 2024 Oct 4;19(10):e0309157. doi: 10.1371/journal.pone.0309157 (PMC11451975; doi:10.1371/journal.pone.0309157)
Supplement: S1 Table — Comparison of time to stability, peak vertical ground reaction force, and time to peak vertical ground reaction forces between conditions (shod and barefoot) and among groups (GYM = gymnasts, CHR = cheerleaders, NAT = non-athletes). Values are mean ± standard deviation. p-values are group main effect, except where specified by a superscript letter which reflect the appropriate pairwise value. (DOCX) [file pone.0309157.s001.docx]

**S1 Table:** **Landing kinetics.** Comparison of time to stability, peak vertical ground reaction force, and time to peak vertical ground reaction forces between conditions (shod and barefoot) and among groups (GYM=gymnasts, CHR=cheerleaders, NAT=non-athletes). Values are mean ± standard deviation. p-values are group main effect, except where specified by a superscript letter which reflect the appropriate pairwise value.

|  |  | GYM | CHR | NAT | Main effect p-value (ω^2^);  pairwise p-value | Condition | Interaction |
| --- | --- | --- | --- | --- | --- | --- | --- |
| Time to Stability (ms) | Barefoot | 311 ± 66 | 345 ± 102 | 415 ± 105 | 0.003*  (0.078);  ^A^0.007,  ^B^0.007 | 0.174 | 0..670 |
|  | Shod | 367 ± 104 | 348 ± 94 | 469 ± 228 |  |  |  |
| pVGRF (BW) | Barefoot | 6.39 ± 0.95 | 5.44 ± 1.13 | 5.31 ± 1.32 | 0.015*  (0.050);  ^A^0.015, | <0.001* | 0.144 |
|  | Shod | 5.20 ± 0.61 | 4.86 ± 0.69 | 4.59 ± 0.54 |  |  |  |
| TTpVGRF (ms) | Barefoot | 53.0 ± 7.5 | 56.6 ± 10.1 | 61.5 ± 9.8 | 0.054*  (0.030);  ^A^0.045 | 0.041* | 0.340 |
|  | Shod | 57.2 ± 4.6 | 58.8 ± 7.3 | 61.8 ± 7.9 |  |  |  |

* denotes significant main effect

^A^ denotes a significant pairwise difference between GYM and NAT

^B^ indicates a significant pairwise difference between CHR and NAT
