# Supplementary material for: Sticking the landing: A comparison of shod vs barefoot landing kinetics and foot muscle characteristics in gymnasts, cheerleaders, and non-athletes
Source: PLoS One. 2024 Oct 4;19(10):e0309157. doi: 10.1371/journal.pone.0309157 (PMC11451975; doi:10.1371/journal.pone.0309157)
Supplement: S2 Table — Comparison of muscle cross-sectional area (CSA) and maximal toe flexion force measurements among groups (GYM = gymnasts, CHR = cheerleaders, NAT = non-athletes). Values are mean ± standard deviation. p-values are group main effect, except where specified by a superscript letter which reflect the appropriate pairwise value. (DOCX) [file pone.0309157.s002.docx]

**S2 Table:** Muscle size and strength. Comparison of muscle cross-sectional area (CSA) and maximal toe flexion force measurements among groups (GYM=gymnasts, CHR=cheerleaders, NAT=non-athletes). Values are mean ± standard deviation. p-values are group main effect, except where specified by a superscript letter which reflect the appropriate pairwise value.

|  |  | GYM | CHR | NAT | Main effect p-value (ω^2^); pairwise p-value |
| --- | --- | --- | --- | --- | --- |
| Muscle CSA  (cm^2) | AH | 2.1 ± 0.5^A^ | 1.9 ± 0.6 | 1.6 ± 0.4 | 0.026* (0.110); ^A^0.02 |
|  | FDB | 1.9 ± 0.3^A^ | 1.7 ± 0.4 | 1.5 ± 0.4 | 0.031* (0.102); ^A^0.024 |
|  | QP | 1.7 ± 0.3^A^ | 1.7 ± 0.5^B^ | 1.2 ± 0.3 | <0.001* (0.276); ^A^0.002, ^B^0.001 |
|  | TP | 3.3 ± 0.5^A^ | 3.1 ± 0.4^B^ | 2.4 ± 0.4 | <0.001* (0.433); ^A^<0.001, ^B^<0.001 |
|  | FL | 4.8 ± 1.1^A^ | 4.2 ± 0.8 | 3.9 ± 0.7 | 0.025* (0.111); ^A^0.026 |
|  | FB | 3.5 ± 0.7 | 3.3 ± 0.6 | 3.1 ± 0.6 | 0.296 (0.010) |
|  | SUM | 17.3 ± 2.2^A^ | 15.9 ± 2.2^B^ | 13.9 ± 1.5 | <0.001* (0.299); ^A^<0.001, ^B^0.021 |
| Maximal Flexion Force (N) | Hallux | 5.3 ± 3.5 | 4.3 ± 1.3 | 3.5 ± 0.9 | 0.091 (0.060) |
|  | Lateral toes | 3.9 ± 1.6 | 4.2 ± 1.4^B^ | 3.0 ± 0.9 | 0.042* (0.091); ^B^0.043 |

* denotes significant main effect

^A^ denotes a significant pairwise difference between GYM and NAT

^B^ indicates a significant pairwise difference between CHR and NAT
